# Red Cabbage Anthocyanin-Loaded Bacterial Cellulose Hydrogel for Colorimetric Detection of Microbial Contamination and Skin Healing Applications

**DOI:** 10.3390/polym17152116

**Published:** 2025-07-31

**Authors:** Hanna Melnyk, Olesia Havryliuk, Iryna Zaets, Tetyana Sergeyeva, Ganna Zubova, Valeriia Korovina, Maria Scherbyna, Lilia Savinska, Lyudmila Khirunenko, Evzen Amler, Maria Bardosova, Oleksandr Gorbach, Sergiy Rogalsky, Natalia Kozyrovska

**Affiliations:** 1 Institute of Molecular Biology and Genetics, National Academy of Sciences of Ukraine, 150, Zabolotnogo Str., 03143 Kyiv, Ukraine; i.e.zaets@imbg.org.ua (I.Z.); t.a.sergeyeva@imbg.org.ua (T.S.); a.v.zubova@imbg.org.ua (G.Z.); v.o.korovina@imbg.org.ua (V.K.); m.v.shcherbyna@imbg.org.ua (M.S.); l.o.savinska@imbg.org.ua (L.S.); 2 Department of Extremophilic Microorganisms Biology, D. K. Zabolotny Institute of Microbiology and Virology of the National Academy of Sciences of Ukraine, 154 Zabolotnogo Str., 03143 Kyiv, Ukraine; o.havryliuk@imv.org.ua; 3 Grup de Biotecnologia Molecular i Industrial, Departament d’Enginyeria Química, Universitat Politècnica de Catalunya (UPC-BarcelonaTech), Rambla de Sant Nebridi 22, 08222 Terrassa, Spain; 4 Institute of Physics, National Academy of Sciences of Ukraine, 46 Nauky Ave., 03680 Kyiv, Ukraine; lukh@iop.kiev.ua; 5 Student Science, s.r.o., č.p. 237, 407 57 Horní Podluží, Czech Republic; 6 Institute of Informatics, Slovak Academy of Science, Dúbravská Str. 9, 84507 Bratislava, Slovakia; maria.bardosova@savba.sk; 7 National Cancer Institute of Ukraine, 33/43, Lomonosova Str., 03022 Kyiv, Ukraine; oleksandr.gorbach@unci.org.ua; 8 V.P. Kukhar Institute of Bioorganic Chemistry and Petrochemistry, National Academy of Sciences of Ukraine, Acad. Kukhar Str. 1, 02094 Kyiv, Ukraine; rogalsky@bpci.kiev.ua

**Keywords:** red cabbage anthocyanins, bacterial cellulose, halochromic pH sensor

## Abstract

Developing innovative, low-cost halochromic materials for diagnosing microbial contamination in wounds and burns can effectively facilitate tissue regeneration. Here, we combine the pH-sensing capability of highly colorful red cabbage anthocyanins (RCAs) with their healing potential within a unique cellulose polymer film that mimics the skin matrix. Biological activities of RCA extract in bacterial cellulose (BC) showed no cytotoxicity and skin-sensitizing potential to human cells at concentrations of RCAs similar to those released from BC/RCA dressings (4.0–40.0 µg/mL). A decrease in cell viability and apoptosis was observed in human cancer cells with RCAs. The invisible eye detection of the early color change signal from RCAs in response to pH alteration by bacteria was recorded with a smartphone application. The incorporation of RCAs into BC polymer has altered the morphology of its matrix, resulting in a denser cellulose microfibril network. The complete coincidence of the vibrational modes detected in the absorption spectra of the cellulose/RCA composite with the modes in RCAs most likely indicates that RCAs retain their structure in the BC matrix. Affordable, sensitive halochromic BC/RCA hydrogels can be recommended for online monitoring of microbial contamination, making them accessible to patients.

## 1. Introduction

Wound healing, as a restoration of skin surface injuries, proceeds quickly and without complications in healthy individuals under sanitary conditions. However, complications during tissue regeneration, such as infections, lead to delays in the healing. Wound skin restoration is a complex, many-stage process involving changes in various biometrics, including pH [1]. Each stage of wound healing is associated with a characteristic local pH in the exudate. The pH changes from 5.4–5.6 in normality to 7.2–8.9 after infection can be successfully detected by pH sensors without wound intervention to conclude the state of the wound bed. Currently, more attention is paid to developing wound dressings, which are used not only to protect the wound and promote tissue regeneration but also to obtain valuable information about the state of wound healing and the treatment prognosis.

Recent advances in novel dressing design have aroused admiration for diverse solutions in the design of intelligent dressings based on modern knowledge of molecular mechanisms of tissue regeneration and advancements in biosensors, nanotechnologies, and 3D printing, among others [2,3,4,5,6]. Wound dressings incorporate various sensors, monitoring conditions in the wound in real time. They may also combine various other techniques, such as photostimulation or electrostimulation, to facilitate personalized wound care [4,7]. Suitable microelectronic wearable sensors have been developed for advanced dressings, enabling real-time monitoring of the fluid within the wound, providing enhanced treatment, thereby revolutionizing conventional healthcare systems [8,9,10]. Nevertheless, it is worth mentioning that the limitations associated with modern therapeutic dressings and their delivery/monitoring systems (all-in-one) are significant. Moreover, advanced strategies are not yet employed in clinics [11].

The public trend of a healthy lifestyle and concern about environmental contamination promotes the selection of natural healing components for wound treatment based on both scientific knowledge and folk experience. Natural biopolymeric formulations, composed of biocompatible, nontoxic, and biodegradable polymers enhanced with phytochemicals, have recently garnered attention for wound care due to their limited adverse effect on health and the environment. Additionally, they enable efficient drug and gene delivery, biosensor incorporation, and in situ diagnostics [11,12,13,14]. Natural, degradable polymeric materials, including chitosan, hyaluronic acid, sodium alginate, gelatin, and carrageenan, were designed to enhance traditional dressings for wound healing by incorporating antimicrobial, anti-inflammatory, and antioxidant ingredients [13,15]. The polymer networks in natural hydrogels form numerous micropores, providing tunnels for a load of therapeutic extracellular vesicles, which regulate inflammation and facilitate vascularization and collagen deposition [16,17].

Compared to other natural wound polymeric dressings, occlusive bacterial cellulose (BC) dressings provide healing in moderate moisture, promoting cell proliferation and tissue granulation and allowing for exudate adsorption and gas exchange—mimicking the extracellular matrix of skin [18,19,20]. Transparent natural BC films enable wound recovery to be monitored without the need for dressing removal. A painless removal of the moist dressing is a valuable advantage of BC dressings, which made BC hydrogels popular in the world wound-dressing market and theaters of combat.

Natural medicinal plant compounds meet the modern requirements of the public and serve as an efficient aid in managing skin problems and as a component of complex healing systems (e.g., hydrogels) due to a set of valuable traits, the absence of side effects, and lower prices for herbal products. Polyphenols, alkaloids, and saponins possess various healthcare effects: anti-inflammatory, antimicrobial, antioxidant, anti-aging, anticancer, etc. [21]. Plant-derived natural compounds also provide efficient, eco-friendly dyes that respond to changes in their surroundings, making them feasible alternatives to toxic and expensive synthetic or inorganic pigments [22]. Among them, flavonoid pigments—anthocyanins (ANCs), a subclass of phenols—are candidates for use as sensors of microbial contamination and wound progression, dependent on pH [23,24,25]. Flavonoid ANCs acquired their antioxidant, anti-inflammation, anticancer, and anti-aging properties [26,27,28,29]. These properties are promising for the efficient wound healing of various etiologies. Highly pigmented red cabbage exhibits mainly cyanidin-3-diglucoside-5-glucoside (Cya-3-diglu-5-glu) and more than 30 different derivatives [30]. Red cabbage ANCs are gaining popularity for utilization due to their availability and low-cost plant resources. However, anthocyanin chromophores degrade at pH values higher than 9 at high temperatures, under exposure to light, or low-dose gamma radiation [30,31]. Nevertheless, methods for improving the pH-sensitive indicator film color sensitivity and stability are being explored [32,33].

In this study, we investigated the biological activities of a bacterial cellulose matrix embedded with red cabbage anthocyanins (RCAs), aiming to design a natural dual-purpose dressing for both healing and monitoring microbial contamination. Our goal was to create a low-cost, easy-to-use, and disposable dressing that is safe and has no adverse effects, in line with the public’s trend towards a healthy lifestyle and concern about environmental contamination

## 2. Materials and Methods

### 2.1. Microorganisms

A kombucha microbial community (KMC) strain, IMBG-1, was used to produce the cellulose-based pellicle film. The medium for KMC growth in static conditions was composed of green leaf tea (0.5%), white sugar (7.0%), and boiled tap water. The KMC was grown within one week, and the cultivation temperature for KMC was 28 °C.

For antimicrobial and color modification research, the strains *Bacillus subtilis* B-901 and *Candida albicans* UCM-1918 (Ukrainian Collection of Microorganisms, Kyiv, Ukraine), *Pseudomonas aeruginosa* ATCC 10145 (American Type Culture Collection, Manassas, VA, USA), *Staphylococcus aureus* ATCC 23235, and *Escherichia coli* ATCC 25922 were used. The cultivation was carried out overnight at 28 °C in Luria–Bertani (LB) broth.

### 2.2. Anthocyanin Extraction

Red cabbage was purchased from the Prague local market in October 2024, and 150 g of plant material was crushed and macerated with 100 mL of distilled water (DW). The pH of the sample was adjusted to 2.0 with 1.0 mol/L hydrochloric acid. The red cabbage anthocyanin was maintained at pH 2, in the dark, and at 4 °C. The prepared extraction was centrifuged at 2000 rpm for 10 min. The supernatant was collected and neutralized with NaOH (2.5 M) to pH 7.0. The obtained extract was filter-sterilized (0.22 µm) and stored in a dark location at 4 °C until use.

#### 2.2.1. Hydrogel Fabrication and Purification

A 4 mm pellicle film was purified as described earlier [6]. Briefly, the BC film was deepened in a 0.5% NaOH solution at 90 °C and stirred for 30 min. Next, the BC film was washed with DW until a pH of 6.0–7.0 was reached. BC films were packed into a bag for protection from external factors, sealed, irradiated by gamma rays at 10 kG at Radma Ltd. (Kyiv, Ukraine), and kept at room temperature.

#### 2.2.2. Quantification of RCA Uptake and Release

Load and release concentrations were found via spectrometric pH-differential method (ND-1000, NanoDrop Technologies Inc, Wilmington, DE, USA) [34]. BC hydrogel films 4 × 4 cm were immersed in 10 mL of RCA extract for loading, and absorption was measured every three hours (n = 3). Cyanidin-3-diglucoside-5-glucoside is a major anthocyanin in the red cabbage extract profile. Therefore, it was taken as a reference compound. The content of anthocyanin monomers was calculated using the following Equation (1) (n = 6):

*MAPC* = (*A* × *MW* × 1000)/(*ε* × *l*); (1)

where *MAPC*—monomeric anthocyanin pigment concentration, *A*—absorption, *MW* = 773.7 g/mol—Cyanidin-3-diglucoside-5-glucoside molecular weight, *ε* = 30,175—L/mol ·cm—C-3-diglu-5-glu molar absorptivity, l = 0.1 cm—spectrophotometer path length.

After that, impregnated films were placed into 10 mL of distilled water. Release concentration was calculated analogously.

The mass fraction of RCAs incorporated into bacterial cellulose (BC) films was determined by gravimetrical analysis, n = 3 [35]. BC films (4 × 4 cm) were gently blotted to remove excess surface moisture and weighed before RCA loading. After immersion in RCA extract, the films were blotted again and reweighed. The samples were then dried at 50 °C until a constant weight, and mass fraction was found via Equation (2). Analogous calculations were made for a wet film (Equation (3)):

(2) $$W=\;\frac{m_{dry\;BC/RCA}-m_{dry\;control}}{m_{dry\;BC/RCA}};$$

(3) $$W=\frac{m_{wet\;BC/RCA}-m_{wet\;BC}}{m_{wet\;BC/RCA}}$$

### 2.3. Anthocyanins’ Hydrogel Characterization

#### 2.3.1. Fourier Transform Infrared Spectroscopy (FTIR)

The FTIR measurements were performed using a Bruker IFS-113v spectrometer (Bruker Corporation, Billerica, MA, USA) at room temperature in the range of 500–4000 cm^−1^ with a spectral resolution of 1.0 cm^−1^ (n = 300). The BC/RCA composite specimens were prepared as described earlier by us (3 × 3 cm, 0.5 mm thick, prepared in triplicate, and dried at 50 °C overnight before analysis) [6]. The RCA extract was smeared as a thin layer on the surface of the KBr plate, which was then dried at 35°C for 24 h.

#### 2.3.2. Scanning Electron Microscopy (SEM)

The BC/RCA hydrogel samples (thickness, 0.5 mm), prepared in triplicate, were dried at 50 °C for 6 h and then coated with gold nanoparticles (10 nm) for SEM analysis. The samples were visualized using a Quanta FEG-250 scanning electron microscope (Model No. 1027641, FEI company, Brno, Czech Republic). The samples were analyzed at a magnification of 90,000 at a voltage of 20 kV. The pore width of the BC and BC/RCA membrane pores (n = 40) was measured by the ImageJ/Fiji^®^ program.

##### Sample Color Determination with Smartphone-Based Sensor System

The sterile BC hydrogel discs (8 mm) were incubated in a 1:1 (*v*/*v*) mixture of the RCA extract and 100 mM phosphate/citrate buffer at varying pH levels (pH 4, 5, 6, and 7) in aseptic conditions for 1 h. Next, the discs were placed on Petri dishes inoculated with bacterial lawns of *B. subtilis* B-901. Color changes in the polymer films were recorded using a Samsung A52 smartphone camera as described earlier [6]. The color intensity and hue changes of the staining of BC hydrogel discs (n = 3) were recorded using the Spotxel Reader 2.5.1 mobile application (SICASYS Software GmbH, Germersheim, Germany).

##### Sample Colorimetric Characterization

Bacterial cellulose hydrogels were cut into circular discs (diameter 2 cm). Red cabbage extract solutions were prepared using phosphate buffer systems adjusted to pH 6.0, 6.2, 6.5, and 7.0. Each disc was immersed in the RCAs corresponding to a specific pH level. After staining, the color difference (ΔE) was measured using a colorimeter (Landtek Distal Colorimeter CM-200S, Guangzhou, China). All experiments were performed in triplicate.

### 2.4. Biological Activities

#### 2.4.1. In Vitro Cell Cultures

Human ATCC MDA-MB-231 (breast adenocarcinoma) cell line was purchased from Sigma-Aldrich (St Louis, MO, USA). Cells were grown in DMEM/F-12 (Sigma-Aldrich, USA) supplemented with 10% fetal calf serum (Sigma-Aldrich, St Louis, MO, USA). The human monocytic leukemia cell line, THP-1, was purchased from Cytion (Eppelheim, Germany) and maintained in RPMI-1640 medium supplemented with 0.1% (*v*/*v*) 2-mercaptoethanol (Thermo Fisher Scientific, Waltham, MA, USA). The cells were cultured at 37 °C in a 5% CO_2_ environment.

#### 2.4.2. Cytotoxicity Assay (Lactate Dehydrogenase Release, LDH)

THP-1 cells (1.5 × 10^4^ cells per mL) were plated in 96-well plates along with treated fresh RCA substances with the following concentrations: 0.2, 2.0, 20.0 µg/mL or doxorubicin (0.5 µg/mL) (positive control) and incubated for 24 h in a 5% CO_2_ at 37 °C. LDH activity was measured in the supernatant using the LDH CytoxTM Assay Kit (426401; BioLegend, San Diego, CA, USA) according to the manufacturer’s protocol and read at 490 nm on a BioRad 680 Plate Reader (Hercules, CA, USA). The percentage of LDH release (cytotoxicity) was calculated in the equation provided by the manufacturer.

#### 2.4.3. Human Cell Line Activation Test (h-CLAT)

The h-CLAT demonstrates CD86 expression in THP-1 cells (sensitization) upon exposure to chemicals [34]. Single-cell suspensions of THP-1 cells were treated with RCAs (4.0 and 40.0 µg/mL), a 1-chloro-2,4-dinitrobenzene (DNCB) (a positive control, 4 µg/mL), or DL-lactic acid (a negative control, 1000 µg/mL). Then, cells were stained with phytoerythrin-conjugated anti-human CD86 antibody (BioLegend, San Diego, CA, USA) and analyzed using a Navios EX Flow Cytometer (Beckman Coulter, Brea, CA, USA) with a 488 nm laser (n = 3). Dead cells were identified using 7-aminoactinomycin D (7-AAD, Beckman Coulter, USA). The relative fluorescent intensity of CD86 higher than 150% was considered positive [36].

#### 2.4.4. Apoptosis Assay

The MDA-MB-231 cells at a density of 1 × 10^6^ cells per well in 6-well plates were treated with two different concentrations of RCA extract (4.0 and 40.0 µg/mL) or doxorubicin (0.5 µg/mL). The treated cells were incubated for 24 h at 37 °C in air with 5% CO_2_ and analyzed using flow cytometry. After treatment with RCAs, cells were suspended in a binding buffer and stained with annexin V-FITC and 7-AAD for 10 min, then analyzed on a Navios EX Flow Cytometer (Beckman Coulter, Brea, CA, USA) (a 488 nm laser) (n = 3).

#### 2.4.5. Antimicrobial Activity

Undecylenic acid (UA, 98%) and diethanolamine (DEA, 98%) were supplied from Sigma-Aldrich (St Louis, MO, USA). To prepare UA/DEA salt, an equimolar mixture of UA and DEA was stirred at 50 °C for 2 h. The salt is a viscous, transparent liquid that is highly soluble in water.

The filter-sterilized RCA samples paired with 2% UA-DEA were allowed to diffuse in sterile 8.0 mm bacterial cellulose hydrogel discs for approximately 1 h and then incubated overnight at 28 °C on microbial lawns prepared on agar plates. Pure cellulose discs served as a negative control. As a positive control, cellulose disks impregnated with UA-DEA (2%) were used. After incubation, clear zones surrounding the discs, corresponding to the antibacterial activity of the tested RCA, were observed. The clear zone was measured in mm (n = 6) using ImageJ 1.54f/Fiji^®^.

### 2.5. Statistics

Three independent measurements were performed for data expressed as means ± standard deviation. A student’s T-test was performed to compare only two groups, and *p*-values less than 0.05 were considered statistically significant. The T-test was conducted under the assumption of normality.

## 3. Results

### 3.1. Red Cabbage Anthocyanin Extract and Impregnated Bacterial Cellulose Hydrogel Films Showed pH-Dependent Colors

**Figure 1:**
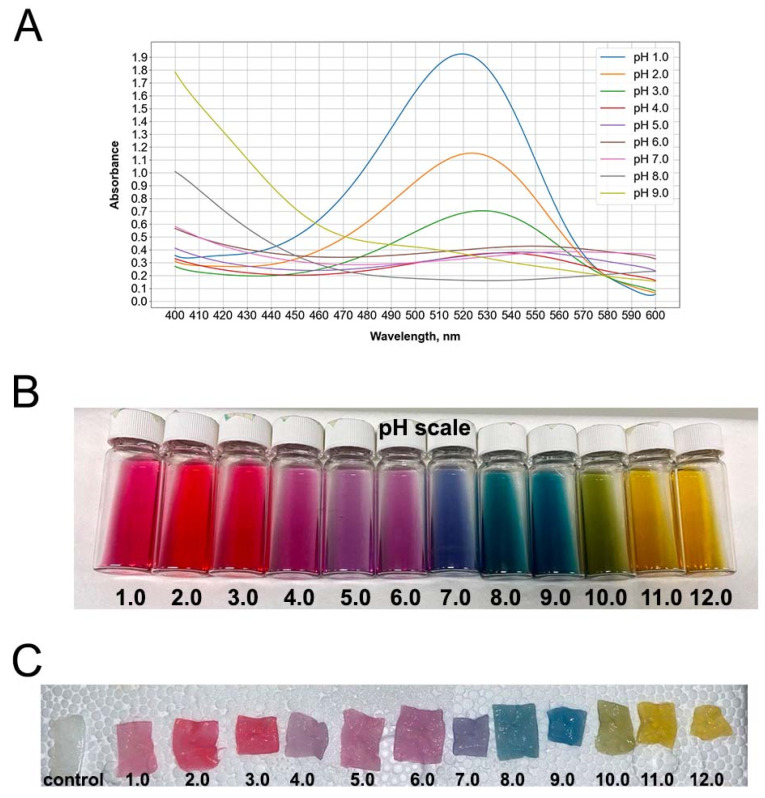
UV–Visible spectra of red cabbage anthocyanin extract at different pH values (**A**); pH-dependent color extracts (**B**) and embedded bacterial cellulose hydrogels (**C**).

The absorbance of pH values in RCAs from 1 to 3 was relatively similar, showing peaks at 520–530 nm for red and pinkish red (Figure 1A,B). An RCA peak was observed at 550 nm within a pH range of 5–6, corresponding to a purple color. pH 7 showed a blue color with an absorbance peak at 565 nm. At pH 8, RCAs exhibited a blue-green color absorbed at 610 nm.

The RCA solution becomes red at pH values lower than 3 because of the flavylium cation presence in the extract. At higher pH values (between 3 and 9), carbinol pseudobases and quinoidal bases are generated, converting the color of the solution from violet to blue. The last species, corresponding to the chalcone pseudobase, turns the solution a yellow color. Analogous colors of RCAs were seen in the impregnated BC matrices (Figure 1C).

The monomeric anthocyanin pigment concentration in RCAs was 401.1 ± 11.4 mg/L, with cyanidin-3-diglucoside-5-glucoside as the major component.

### 3.2. A Color Level Visualization and Its Measurement

#### 3.2.1. RCA-Impregnated BC Films for the Control of Bacterial Contamination

**Figure 2:**
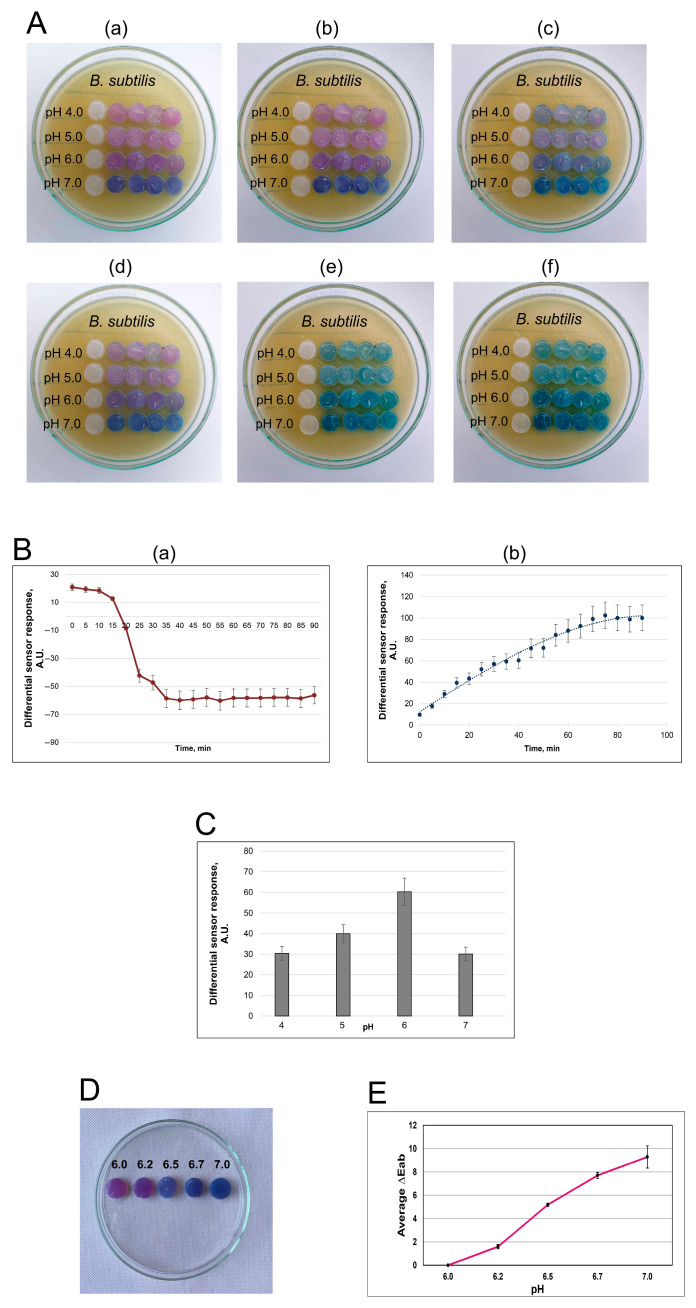
Smartphone-based system for BC/RCA film color determination (**A**–**C**). A, color changes of the KMC films impregnated with natural anthocyanins from red cabbage depending on time of the contact with *B. subtilis* lawn:—0 min (**Aa**), 10 min (**Ab**), 20 min (**Ac**), 40 min (**Ad**), 90 min (**Ae**), and 120 min (**Af**) and pH of the buffer solution used for the immobilization. (**B**), typical calibration curves of the smartphone-based Sensor based on KMC films impregnated with anthocyanins of red cabbage (*Brassica oleracea var. capitata f. rubra L*). Dependence of the red (**Ba**) and blue (**Bb**) color intensity of the BC films with immobilized RCAs at the time of their contact with *B. subtilis* lawns. (**C**), typical values of the sensor responses on the pH of the buffer used for RCA immobilization. The contact time between the BC/RCA films and the *B. subtilis* lawns was 40 min. The colorimetric response of bacterial cellulose discs stained with RCAs was recorded across a pH from 6.0 to 7.0 (**D**,**E**). (**D**), a gradual shift in BC/RCA films’ coloration from pink (pH 6.0) to blue (pH 7.0). (**E**), the average ΔEab within a pH 6.0–7.0, with the most significant perceptible changes occurring beyond pH 6.5.

The BC films were impregnated with RCAs dissolved in 100 mM phosphate-citrate buffer at pH values of 4.0–7.0. The BC/RCA films were demonstrated to change their staining from red-violet to blue-green color starting from the first minutes of their contact with the *B. subtilis* lawn (Figure 2).

It has been demonstrated that the color changes can be both visually observed (Figure 2A) and easily registered using the Spotxel Reader 2.2.3 smartphone application (SICASYS Software GmbH, Germany) (Figure 2B). Importantly, the intensity of the blue-green staining visually does not increase after 60 min of incubation (Figure 2(Bb)), while the intensity of the red staining sharply decreases during the first 30 min and remains constant thereafter (Figure 2(Ba)). Selective elements of the developed sensor based on BC films impregnated with RCAs at a pH 6.0 value were shown to be the most effective for controlling bacterial metabolite activities (Figure 2A). At the same time, BC/RCA films at pH 4.0, pH 5.0, and pH 7.0 demonstrated significantly higher differential sensor responses (an increase in the blue color intensity) than the BC films impregnated with RCAs at pH 6.0 (Figure 2C).

#### 3.2.2. Colorimetry of BC/RCA Films

The colorimetric response of bacterial cellulose discs stained with RCAs was evaluated across a pH range from 6.0 to 7.0. Visual inspection revealed a gradual shift in coloration from pink (pH 6.0) to blue (pH 7.0), as shown in Figure 2D. However, the intermediate shades observed at pH 6.2 and 6.5 were subtle and complex, making them difficult to distinguish with the human eye. Only the extreme pH points (6.0 and 7.0) demonstrated visibly distinct hues, while the transitions between adjacent pH values (especially between 6.2 and 6.5) appeared visually similar, posing a challenge for accurate color assessment without instrumentation.

To address this limitation, quantitative color difference (ΔEab) values were obtained using a colorimeter (Figure 2E). The ΔEab values increased progressively with rising pH, confirming that measurable chromatic changes occurred even when visual differences were minimal. Specifically, the average ΔEab rose from 0.2 ± 0.1 at pH 6.2 to 9.1 ± 1.2 at pH 7.0, with the most significant perceptible changes occurring beyond pH 6.5.

### 3.3. Quantification of RCA Uptake and Release

**Figure 3:**
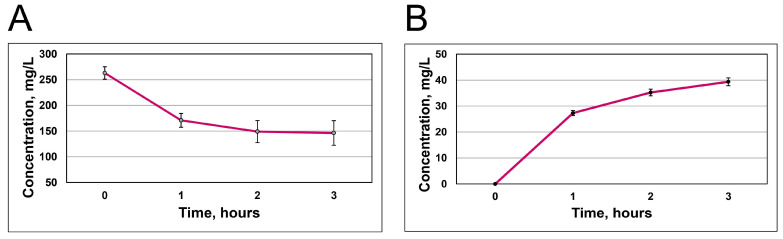
The absorbance of red cabbage anthocyanin extract by a hydrogel film (**A**) and the anthocyanin extract released from the hydrogel into distilled water (**B**).

The load of RCAs into BC seems to peak at 2 h. At this point, the BC hydrogel film absorbs around 117 ± 36 mg/L (Figure 3A). Then, the diffusion process between the solution and the BC hydrogel pores starts. Following three hours, BC/RCA released 39.4 ± 1.5 mg/L of anthocyanins into 10 mL of DW (Figure 3B).

The mass fraction of RCAs incorporated into BC films averaged 0.36 ± 0.04 g/g relative to the dry weight of the cellulose matrix. When calculated based on the wet weight of the loaded films, the corresponding value was 0.46 ± 0.02 g/g.

### 3.4. Biological Activities Characterization

#### 3.4.1. Antimicrobial Activity

**Figure 4:**
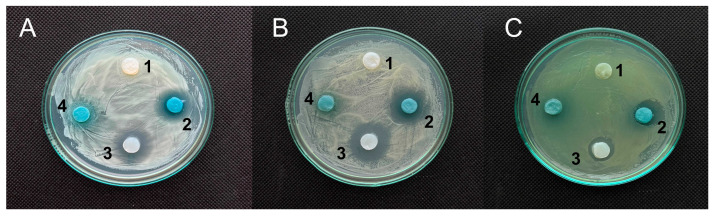
Bacterial cellulose (BC)-based hydrogel discs (1) impregnated with RCAs (4) and UA-DEA salt (3) and BC/RCA/UA-DEA (2) were placed on the lawns of (**A**) *Staphylococcus aureus*; (**B**) *Candida albicans*; and (**C**) *Pseudomonas aeruginosa* and incubated for 16 h. As controls, pure hydrogel (1) and hydrogels with UA-DEA (2%) (3) were used.

RCA/BC samples showed low antimicrobial activity against *S. aureus, C. albicans*, and *P. aeruginosa* strains. However, RCA/BC samples paired with UA-DEA salt (2%) showed a more pronounced effect against the mentioned strains, with clear halos of 18.9 mm ± 0.4; 17.0 ± 3.8 mm; and 13.8 ± 1.4 mm, respectively (Figure 4A–C). The results indicate that undecylenic acid, a natural unsaturated fatty acid, can be successfully used in its water-soluble form, UA-DEA as a mild antimicrobial additive in RCA/BC-based wound dressing materials. Considering the nontoxicity and biodegradability of UA-DEA salt, as well as its excellent compatibility with BC matrix, the high loading of this soft antimicrobial agent in RCA/BC composites can be realized.

#### 3.4.2. Cytotoxicity Assays

##### No Cytotoxicity via LHD Release in Leukemic Monocytes THP-1

**Figure 5:**
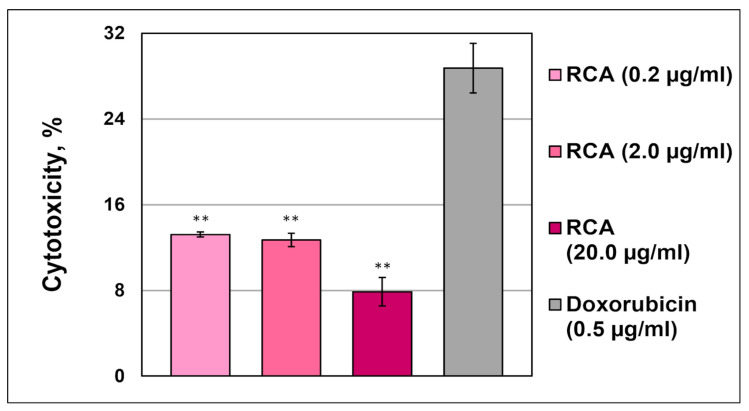
The levels of cytotoxicity in THP-1 cells treated with red cabbage anthocyanins. As a positive control, doxorubicin (0.5 µg/mL) was used. n = 6. **—indicates *p*-value < 0.001.

LDH activities in the THP-1 cell culture supernatant were 2–3-fold lower than in the positive control when anthocyanins at a concentration even higher than that potentially released from halochromic cellulose-based films (4.8 µg/mL) were used. The LDH release of THP-1 cells induced by RCAs was dose-dependent (Figure 5). This finding demonstrated that red cabbage anthocyanins did not damage cell membranes and, therefore, did not exhibit cytotoxicity in leukemic monocytes.

#### 3.4.3. h-CLAT

**Figure 6:**
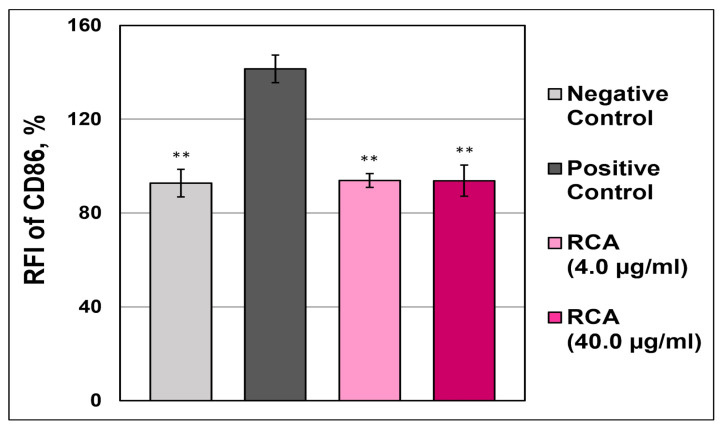
The relative fluorescent intensity (RFI) of the cluster of differentiation CD86 of human monocytic THP-1 cells treated with red cabbage anthocyanins (RCAs), a 1-chloro-2,4-dinitrobenzene (DNCB), or lactic acid. The skin-sensitizing potential of RCAs was determined using flow cytometry analysis of THP-1 cells treated with RCAs (4.0 and 40.0 µg/mL), DNCB (10 µg/mL), or lactic acid (1000 µg/mL) using phycoerythrin-conjugated anti-human CD86 antibody. n = 3. **—indicates *p*-value < 0.001.

To examine the skin-sensitizing potential of anthocyanins from red cabbage, we performed an h-CLAT assay [36] using THP-1 cells. In Figure 6 we demonstrate that 0.4 and 40.0 μg/mL RCAs did not increase the RFI of CD86 higher than 150%, which is the critical cut-off value for potential sensitizers (Figure 6). This result indicates that red cabbage anthocyanins have no potency to cause skin sensitization. In contrast, cells treated with positive control demonstrated 150% RFI (cell sensitization) and death in 45% of the cells.

#### 3.4.4. Apoptosis in Human MDA-MB-231 In Vitro Cells

**Figure 7:**
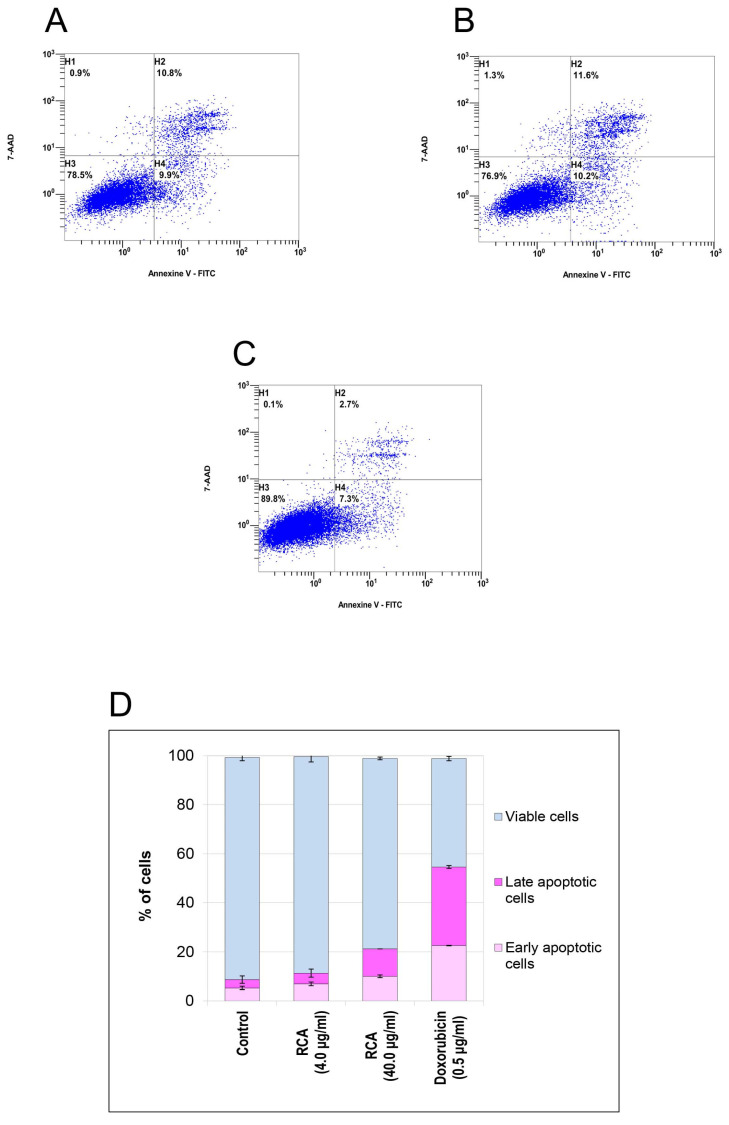
Apoptosis of human MDA-MB-231 in vitro cells treated with anthocyanins extracted from red cabbage (RCA) (4.0 and 40.0 µg/mL) (**A**), doxorubicin (0.5 µg/mL) (**B**), and untreated cells (**C**) was determined by flow cytometry. The apoptosis analysis of MDA-MB-231 cells revealed viable cells, characterized by low FITC and low 7-AAD signals (quadrant H3); apoptotic cells, marked by high FITC and low 7-AAD signals (quadrant H4); and secondary necrotic cells, identified by high FITC and high 7-AAD signals (quadrant H2). Dependence of early and late apoptosis MDA-MB-231 cell rate on the treated RCA concentration (**D**).

The rate of early and late apoptosis was evaluated using flow cytometry. The biparametric histogram shows three distinct populations of in vitro cells: (1) viable cells, which had low FITC and low 7-AAD signal (quadrant H3); (2) apoptotic cells, which had high FITC and low 7-AAD signal (quadrant H4) (early apoptosis); and (3) secondary necrotic cells, which had high FITC and high 7-AAD signal (quadrant H2) (late apoptosis) (Figure 7A–C). The results presented in Figure 7A,D, demonstrate that the early apoptotic cells increased after RCA treatment, compared with the PBS (negative control) (Figure 7C), but did not exceed the positive control cells (Figure 7B).

### 3.5. Physicochemical Characterization

#### 3.5.1. Scanning Electron Microscopy

**Figure 8:**
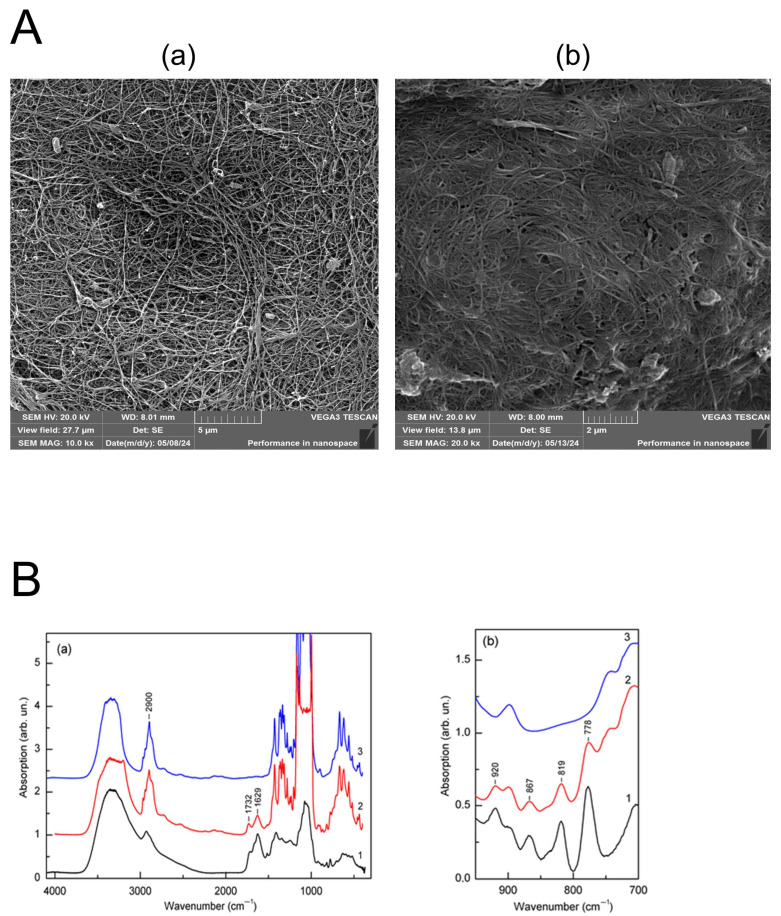
Physical characterization of bacterial cellulose matrices impregnated with red cabbage anthocyanins (RCA) with SEM and FTIR analyses. (**Aa**), an SEM image of the purified BC’s nanostructure. Scale bar: 5 μm. (**Ab**), an SEM micrograph of the BC loaded with RCA. Scale bar: 2 μm. The mean width for BC pores is 1,5 ± 0,49 µm; the mean width for BC/RCA pores is 350 ± 15 nm. Mean values are significantly different (*p* < 0.05). (**Ba**), the FTIR spectra of BC/RCA. Accordingly, 1, 2, 3—spectra for RCA, BC/RCA, and BC. (**Bb**), a fragment of the absorption spectrum, where characteristic modes of anthocyanins are recognized.

The RCAs altered the morphology of the fibrils in the cellulose matrix after impregnation, as shown in Figure 8(Ab). In particular, the width of the BC/RCA pores decreased significantly from 1.5 ± 0.49 µm (control BC) to 350 ± 15 nm. The fibrillar network was denser in the BC/RCA film, possessing rare pores. In contrast, the SEM micrographs of the purified BC showed an extensively entangled fibril network with an irregular fibril void arrangement (Figure 8(Aa)).

#### 3.5.2. FTIR Spectroscopy

The absorption spectra obtained for the original cellulose, RCAs, and the cellulose/RCAs are shown in Figure 8(Ba). The upload of RCAs to cellulose increased the hydroxyl content in the sample, which was manifested in the broadening of the O-H-related stretching vibrational mode toward low frequencies, which coincided with the corresponding broadening of the OH-related band in RCA and led to the appearance of several additional vibrational absorption components in the spectra: at 1629 and 1732 cm^−1^, which coincide with the vibrational modes of RCA. The band at 1629 cm^−1^ can be attributed to the aromatic C=C stretching of alkenes, and the band at 1732 cm^−1^ is characteristic of the C=O stretching of polyphenols [37]. In the BC/RCA, the appearance of new vibrational modes with maxima at 920 cm^−1^ (C=C bending), 867 and 778 cm^−1^ (C-H bending of aromatic hydrocarbons in 6-membered aromatic rings), and 819 cm^−1^ (C-O-C stretching, symmetric vibrations) was observed, which coincided in position with the corresponding vibrational modes in RCAs (Figure 8(Bb)). Spectral analysis also showed that the RCA concentrations used had no noticeable effect on the presence, energy position, and relative intensities of vibrational modes characteristic of cellulose. There was also no effect of the additive on the vibrational mode at 1430 cm^−1^, which characterizes the crystallinity of cellulose, and the mode at 898 cm^−1^, which characterizes its amorphousness. The LOI (Lateral Order Index), which was defined as the ratio between the intensity of the bands at 897 cm^−1^ (glycosidic bond β-(1,4) in cellulose) and 1430 cm^−1^, for both BC (0.120) and BC/RCA (0.116), has no statistically proven difference. The TCI (Total Crystallinity Index), estimated on the intensities of the absorption components (I372/2900), decreased by 2% in the BC/RCA film from 71% to 69%, which was not statistically significant.

## 4. Discussion

Here, we combined the pH-sensor capability of anthocyanins extracted from highly colorful red cabbage with their healing potential in skin wound dressings. Previously, red cabbage extracts were mainly incorporated into polymer materials for pH sensing in package films designed for food freshness preservation, e.g., within bacterial cellulose [31,38], bacterial cellulose nanofiber/gelatin film [39], or vegetative nanocellulose [40].

With the aim of their use in wound management, RCAs have been incorporated into textile polymers to develop pH-responsive smart films, such as electrospun cellulose acetate nanofiber material [41]. In such cases, anthocyanin dyes require a mordant in textile fibers due to their limited stability [42]. However, a mordant reduces the dye’s solubility in water, preventing its release from the matrix, which limits the beneficial healing. Existing cellulose diagnostic sensors exhibit alterations in the affected medium color, and they are promising for various types of microbial presence diagnostics. Nevertheless, their use has some restrictions in wound therapy, e.g., due to the presence of metal or other mordants.

Our results demonstrate that the presence of a small RCA mass fraction in BC can be used as a pH sensor, indicating a color change within a few minutes after contact with microbial metabolites. Anthocyanins changed color within the BC matrix quickly, and their color was visible to the naked eye. The color change signal from RCAs in response to pH, which was invisible to the eye, was evaluated using a colorimeter or a smartphone-installed application.

In this study, we suggest that anthocyanins extracted from red cabbage possess a healing potential, likely due to their antimicrobial, antioxidant, anti-inflammatory, anticancer, and senolytic properties. First, we used a hydrated bacterial cellulose matrix, a polymer that possesses open functional groups, to hijack anthocyanins. This affordable material, due to the reactive hydroxyl groups in the BC structure, can be subjected to various in vivo and in vitro modifications for the development of functionalized BC on demand of wound or burn management specificity [43,44] or other biomedical applications (controlled drug delivery, skin cancer treatment, scaffold manufacture, etc.) [17,45]. Hydrated BC, a matrix for therapeutic agents, is an excellent dressing for covering wound surfaces and thermal skin burns, providing successful healing of injured skin in moderately wet conditions without scar formation [19]. The BC polymer structure potentially provides a hub for integrating sensing elements into BC films. In our previous project, we demonstrated that anthocyanins from elderberry fruits modified cellulose polymers, forming weak connections with OH groups [6]. In this project, anthocyanins were physically absorbed into the porous BC matrix, rather than covalently bound. The observed thickening and densification of microfibrils in SEM images reflect structural morphological changes caused by penetration and retention of RCAs within the BC network, rather than chemical substitution. However, the structure of BC polymer was not modified, which might be explained by the unchanged rate of its crystallinity, i.e., nonsignificant change on the vibrational mode at 1430 cm^−1^, which characterizes the crystallinity of cellulose, and the mode at 898 cm^−1^, which characterizes its amorphousness. Additionally, the LOI has not undergone significant changes. This indicates that the RCA additive did not alter the structure of cellulose. The complete coincidence of the vibrational modes detected in the spectra of the BC/RCA composite with the modes in RCAs most likely indicates that RCAs retained its structure in the BC matrix but did not impact the polymer structure and provide an easy release of anthocyanins into the wound. A mass fraction of RCAs in BC (0.36 ± 0.04 g/g), registered by FTIR and released from BC/RCA, can induce apoptosis in cancer cells.

Secondly, we attempted to show that BC impregnated with RCAs had the potential for skin healing based on our current knowledge and the available literature. We know that various anthocyanin pigments have demonstrated the ability to protect against a myriad of human diseases. The colorful anthocyanins are the most recognized phytochemicals with free-radical scavenging and antioxidant capacities associated with disease healing [27,46,47]. Due to biological activities, anthocyanins also protect against DNA cleavage, boost cytokine production, induce regulated cell death [48], exhibit antimicrobial [49], anti-inflammatory [26], anticancer [50,51], and senolytic activities [52]. A potential for wound healing was demonstrated for elderberry anthocyanin crude extract in in vitro experiment using murine fibroblasts [6].

Our results on the biological activities of RCAs in the matrix of bacterial cellulose showed that there was low-level LDH activity/cytotoxicity towards the monocytic leukemia cell line THP-1, as well as an absence of proinflammatory cytokine boosting, e.g., IL-1β, characteristic of pyroptosis. We used the human cell line activity test as a method, which describes the change in the expression of the cell surface marker CD86 associated with the process of monocyte activation and a transition to dendritic cells following exposure to sensitizers. This test revealed no significant expression of the cell surface marker CD86, indicating the activation of dendritic cells, but no skin-sensitizing potential.

Several studies have investigated the mechanisms of RCA anticancer activities in vitro [53,54,55,56] and in vivo in animal models [57]. Scholars have identified several key mechanisms, including the following: increased expression of cytokines, such as TNF-α [51]; caspase-dependent and caspase-independent apoptosis; cell cycle arrest in the G0/G1 phase [52,53]; altered lipid metabolism in tumors [55]; and others. Many plant species’ anthocyanins have anti-melanoma activity [58,59]. The anti-melanoma mechanisms of anthocyanin extract are attributed to cell cycle arrest and induction of apoptosis, accompanied by the upregulation of caspase-8 and p53 expression [60]. BC/ANC hydrogels might be used as an adjuvant in skin cancer therapy.

Our attention was focused on ANC’s capacity to mitigate skin problems as ANCs reduce reactive oxygen species, activate ERK1/2 and CREB signaling pathways, and protect against regulated cell death, upregulating Bcl-2 expression and downregulating caspase-3 expression [61]. Research data show that, beginning from cell cultures and animal models to clinical studies, ANCs not only reduce oxidative stress and DNA damage but also alleviate the inflammatory response and promote collagen synthesis [62,63]. Anthocyanins promote the apoptosis of senescent cells, leading to the attenuation of aging in in vitro experiments, and have anti-aging potential for the skin [64]. These valuable characteristics of ANCs may also be utilized as cosmeceuticals due to the absence of toxicity. Therefore, anthocyanin hydrogels can be recommended for the transdermal delivery of ANC extract in post-operative care, after the pilling procedure, and for pigmentation removal.

Additionally, the stability of RCAs within the BC matrix over time, under varying physiological conditions, and during sterilization procedures will ultimately be the responsibility of the manufacturer, alongside considerations such as shelf life and storage conditions. Given the likely small-scale production and point-of-use scenario, a practical approach could involve the use of individually packaged sterile BC films and sterile RCA extract, combined ex tempore before application. This format minimizes degradation risk, supports sterility, and remains functionally adequate for the intended short-term contact with wounds. The use of low-cost, non-toxic raw materials for halochromic cellulose-based dressings also supports the future development and optimization of biotechnological parameters, including those mentioned above, required for scaled production.

Overall, our study highlights the potential of the BC/RCA combination as a promising dual dressing for wound and burn care, as well as online monitoring of the healing process. In the future, our research will focus on in vivo experiments devoted to the transdermal delivery of RCAs in complementary skin senescence-targeted therapies for the treatment of chronic wounds.

## 5. Conclusions

In natural hydrogels, their polymer networks form numerous micropores, providing tunnels for a load of therapeutics, thus making such polymers promising for wound healing. Among them, occlusive bacterial cellulose films mimic the skin extracellular matrix and can be used for embedding both pH-sensing and healing agents. Natural medicinal plant compounds, such as anthocyanins, serve as natural pH indicators and meet the requirements of skin wound management due to their healing potential and lack of side effects. A smart hydrogel dressing prototype, combining bacterial cellulose polymer and red cabbage anthocyanins, has been designed for colorimetric testing of wound microbial contamination, enabling in situ control or remote online monitoring with an application installed on a smartphone. The dual-purpose hydrogel dressing does not exhibit cytotoxicity or cell sensitization in vitro on human cells. The BC/RCA hydrogel demonstrates clear potential as a low-cost, biodegradable material for wound status monitoring and healing support and will be further validated under real-use conditions.

## Data Availability

Data supporting reported results are present in the manuscript.
